# Comparative Evaluations of the Pathogenesis of Candida auris Phenotypes and Candida albicans Using Clinically Relevant Murine Models of Infections

**DOI:** 10.1128/mSphere.00760-20

**Published:** 2020-08-05

**Authors:** Taissa Vila, Daniel Montelongo-Jauregui, Hussian Ahmed, Taanya Puthran, Ahmed S. Sultan, Mary Ann Jabra-Rizk

**Affiliations:** a Department of Oncology and Diagnostic Sciences, School of Dentistry, University of Maryland, Baltimore, Maryland, USA; b Department of Microbiology and Immunology, School of Medicine, University of Maryland, Baltimore, Maryland, USA; University of Georgia

**Keywords:** fungal pathogens, *Candida auris*, *Candida albicans*, animal models, biofilm formation, biofilms, host-pathogen interactions

## Abstract

The newly emerged *Candida* species Candida auris is associated with an exponential rise in life-threatening invasive disease in health care facilities worldwide. Unlike other species, C. auris exhibits a high level of transmissibility, multidrug resistance, and persistence in the environment, yet little is known about its pathogenesis largely due to limited data from animal models. Based on *in vitro* biofilm evaluations and confocal laser scanning microscopy, C. auris phenotypes with different biofilm-forming abilities were identified, indicating potential clinical implications. Using clinically relevant murine models of implanted catheter, oral, and intraperitoneal infections, we comparatively evaluated the host site-specific pathogenic potential of C. auris phenotypes and Candida albicans. Based on the results of microbial recovery and scanning electron microscopy analysis of explanted catheters, compared to C. albicans, C. auris more avidly adhered and formed biofilms on catheters. However, although C. auris adhered to oral tissue *ex vivo*, unlike C. albicans, it failed to colonize the oral cavity *in vivo*, as demonstrated by microbial recovery and tissue histopathology analysis. In contrast, recovery from peritoneal lavage fluid and kidneys during time course experiments demonstrated that C. auris persisted longer in the peritoneal cavity and kidneys. Although there were clear niche-specific differences in pathogenic features between C. auris and C. albicans, no significant differences were noted between the C. auris phenotypes *in vivo*. The combined findings highlight unique niche-specific pathogenic traits for C. auris warranting further investigations. Understanding the factors contributing to the rise of C. auris as a nosocomial pathogen is critical for controlling the spread of this species.

**IMPORTANCE** The newly emerged *Candida* species C. auris has been associated with an exponential rise in invasive disease in health care facilities worldwide with a mortality rate approaching 60%. C. auris exhibits a high level of transmissibility, multidrug resistance, and persistence in hospital environments, yet little is known about its pathogenesis largely due to limited data from animal studies. We used clinically relevant murine models of infection to comparatively evaluate the host niche-specific pathogenic potential of C. auris and C. albicans. Findings demonstrated that C. auris adheres more avidly, forming robust biofilms on catheters implanted in mice. However, although C. auris adhered to oral tissue *ex vivo*, it failed to colonize the oral cavity *in vivo*. In contrast, in the intraperitoneal infection model, C. auris persisted longer in the peritoneal cavity and kidneys. Understanding the host-pathogen factors contributing to the rise of C. auris as a nosocomial pathogen is critical for controlling the spread of this species.

## Abstract (precis)

The newly emerged *Candida* species C. auris has been associated with an exponential rise in invasive disease in health care facilities worldwide with a mortality rate approaching 60%. C. auris exhibits a high level of transmissibility, multidrug resistance, and persistence in hospital environments, yet little is known about its pathogenesis largely due to limited data from animal studies. We used clinically relevant murine models of infection to comparatively evaluate the host niche-specific pathogenic potential of C. auris and C. albicans. Findings demonstrated that C. auris adheres more avidly, forming robust biofilms on catheters implanted in mice. However, although C. auris adhered to oral tissue *ex vivo*, it failed to colonize the oral cavity *in vivo*. In contrast, in the intraperitoneal infection model, C. auris persisted longer in the peritoneal cavity and kidneys. Understanding the host-pathogen factors contributing to the rise of C. auris as a nosocomial pathogen is critical for controlling the spread of this species.

## INTRODUCTION

Candida auris has inexplicably and simultaneously emerged on six different continents as a nosocomial pathogen causing outbreaks in health care facilities in more than 40 countries (1–5), with mortality rates as high as 60% (6, 7) (https://www.cdc.gov/fungal/diseases/candidiasis/tracking-c-auris.html). C. auris exhibits several concerning features compared to other *Candida* species; the most surprisingly feature is the efficient person-to-person transmission (8). Typically, infections caused by *Candida* arise from the patient’s own microbiome; however, there is no evidence that C. auris can colonize the gastrointestinal tract or the oral cavity (9, 10). In addition to its aptitude to colonize skin, survive for weeks on nosocomial surfaces, and resist common disinfectants, C. auris exhibits high levels of drug resistance. More concerning however, is its unique ability to develop resistance to all main classes of antifungals (azoles, polyenes, echinocandins), severely limiting treatment options (7, 10, 11). In fact, the multidrug resistance pattern has been observed in around 40% of clinical isolates (7, 10). Compounding its high transmissibility and multidrug resistance, misidentification by available systems has resulted in delay in detection, further complicating clinical management (12).

While much remains unknown about its biology, *in vitro* studies demonstrated that C. auris expresses several key virulence factors common to *Candida*, such as phospholipases, proteinases, secreted aspartic proteases, adhesins, and the ability to form biofilms (13–16). One interesting observation is an aggregative phenotype where daughter cells fail to be released after budding, causing isolates to grow in clumps (14, 17, 18). This aggregative form was shown to be linked to transcriptional changes in genes involved in cell adhesion to surfaces (1), inducible by exposure to antifungals *in vitro* (19), and less virulent than the nonaggregative phenotype in invertebrate infection models (14, 17). Although yet to be investigated in vertebrate animal models, this unique growth feature could have potential clinical implications.

Unlike Candida albicans, C. auris does not undergo morphological switching between yeast and hyphal forms (5, 17), and the lack of filamentation may explain the distinct niches of colonization between the species; while C. albicans can colonize and infect mucosal surfaces, C. auris primarily colonizes skin (13, 16, 20, 21). However, occasional elongation of cells in C. auris into a filamentous and/or pseudohyphal form has been reported in response to temperature, cell cycle arrest, or depletion of Hsp90 (22, 23) and DNA damage from exposure to antimicrobial agents (24). Although C. auris does not form true hyphae, it can adhere and form robust biofilms on surfaces, albeit the biofilms are less complex than those formed by the hypha-producing C. albicans (13, 14, 25). Biofilm formation accounts for much of the antifungal tolerance among *Candida* species as a result of drug sequestration by the biofilm mannan-glucan polysaccharide matrix (26). While the molecular mechanisms governing drug resistance in C. auris are not fully characterized, a recent study demonstrated that the extracellular polysaccharide biofilm matrix sequestered nearly 70% of available triazole drug *in vitro* (27), clearly indicating a key role for C. auris biofilm formation in its high level of drug tolerance (7, 9, 25, 27).

Despite the gravity of this newly emerged nosocomial pathogen, few *in vivo* models of infections have been used to evaluate C. auris, and thus, a great deal is yet to be understood as to how C. auris colonizes and causes disease. Moreover, although indwelling venous catheters are considered important predictors of C. auris infections in hospitals, *in vivo* biofilm-associated infections remain understudied. Similarly, the ability of C. auris to colonize oral mucosal surfaces has not been observed clinically or investigated. Therefore, reliable animal models for C. auris candidiasis are critical to study the unique aspects of C. auris pathogenesis and host-pathogen interaction. To that end, in this study, murine models of oropharyngeal, intraperitoneal, and implanted catheter infections were used to comparatively investigate the pathogenic potential of two phenotypically different C. auris isolates. Importantly, to fully characterize the pathogenesis of C. auris, C. albicans, the species considered most pathogenic, was also included.

## RESULTS

### Strain-dependent variations in C. auris biofilm formation.

**FIG 1:**
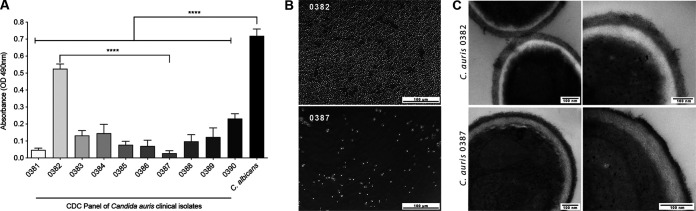
Comparative evaluation of biofilm formation by C. auris clinical isolates and C. albicans. (A) Quantitative evaluation of C. auris and C. albicans *in vitro* biofilm formation. Based on measurement of the metabolic activity of biofilms grown for 24 h on polystyrene microplates, compared to C. albicans, all C. auris isolates exhibited reduced biofilm formation; however, a wide range of biofilm-forming abilities was noted among the isolates with isolate 0382 showing the highest activity and isolate 0387 showing the lowest activity. OD, optical density. Values are means plus standard errors of the means (error bars). ****, *P* < 0.0001. (B) Bright-field microscopy of C. auris 0382 and 0387 24-h biofilms. (C) Transmission electron microscopy analysis of cell wall structure of biofilm-associated cells of both C. auris isolates indicated differences in the outer fibrillar layer with that of 0382 appearing longer compared to the shorter but more dense 0387.

Quantitative evaluation of biofilms demonstrated that all C. auris isolates formed significantly (*P* < 0.0001) less biofilms compared to C. albicans (Fig. 1A). However, there were significant variations among the C. auris isolates, with isolates 0382 and 0387 being the most and least efficient, respectively (Fig. 1A). Microscopic images revealed a dense hyphal biofilm for C. albicans; for C. auris isolates, biofilms consisted of yeast cells, but the architecture differed vastly with some appearing homogeneous covering the whole surface (including isolate 0382), and some consisting of sporadic cell clusters (including isolate 0387) (Fig. 1B; see also Fig. S1 in the supplemental material). Based on the preliminary screening, two representative isolates were selected for further analysis, with isolate 0382 designated a “high biofilm former” (HBF) and isolate 0387 designated a “low biofilm former” (LBF).

10.1128/mSphere.00760-20.1 FIG S1 Bright-field images of biofilms formed by C. auris isolates (CDC panel). Isolates 0382 and 0387 were designated the highest and lowest biofilm formers, respectively. Bar represents 100 μm. Download FIG S1, TIF file, 1.0 MB. Copyright © 2020 Vila et al. 2020 Vila et al. This content is distributed under the terms of the Creative Commons Attribution 4.0 International license.

### Confocal laser scanning microscopy (CLSM) reveals variable spatial biofilm distribution.

**FIG 2:**
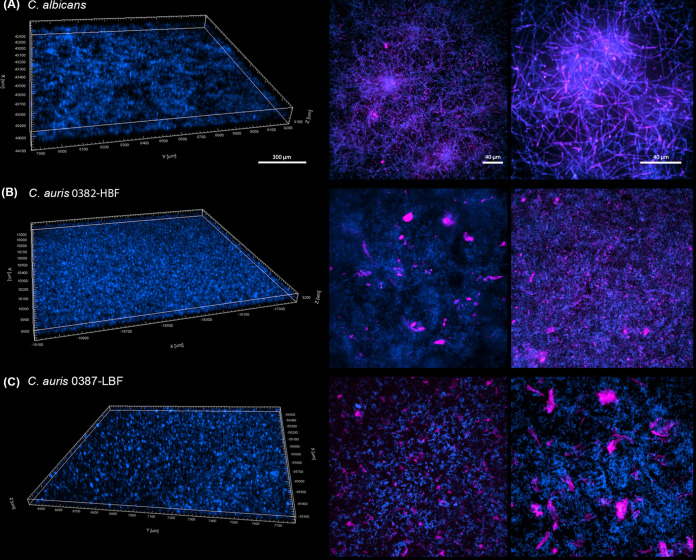
Confocal laser scanning microscopy evaluation of 24-h-grown biofilms formed by the two C. auris phenotypes (0382-HBF and 0387-LBF) and C. albicans. (A) C. albicans; (B) C. auris 0382-HBF; (C) C. auris 0387-LBF. (Left) Z-stack reconstructions of biofilm structure demonstrating a thicker biofilm for C. albicans; the biofilm of isolate 0382-HBF appeared homogeneous and more tightly packed than that formed by isoalte 0387-LBF, which appeared patchy and heterogeneous. (Right) Confocal reconstructions showing biofilm structure and extracellular matrix distribution. C. albicans biofilm exhibited complex architecture consisting of hyphal (blue) and extracellular polysaccharides (fuchsia). (B and C) Significant differences in architecture of C. auris isolates consisting of yeast cells (blue) with a marked presence of secreted polysaccharides (fuchsia). Cell wall chitin stained with calcofluor white (blue) and cell wall and extracellular matrix polysaccharides stained with concanavalin A (fuchsia) are apparent.

Based on fungal cell wall and mannan and glucan secreted polysaccharide fluorescent staining, C. albicans images revealed a dense biofilm with complex architecture consisting of hyphal and extracellular polysaccharides with significantly less dense biofilms formed by the C. auris isolates. (Fig. 2A). However, biofilm of isolate 0382-HBF appeared homogeneous and more tightly packed than that formed by isolate 0387-LBF, which appeared patchy and heterogeneous (Fig. 2A to C).

### Transmission electron microscopy (TEM) indicates variability in cell wall-outer fibrillar layer density.

The overall cell structures of C. albicans and C. auris were comparable consisting of multilayer cell wall with an outer fibrillar layer (Fig. S2). However, noticeable differences in fibrillar layer density and thickness were observed between the C. auris isolates in biofilm-associated (Fig. 1C), as well as planktonic cells (Fig. S2), with 0382-HBF cells exhibiting a longer fibrillar layer compared to the shorter but more dense layer seen in 0387-LBF cells (Fig. 1C and Fig. S2).

10.1128/mSphere.00760-20.2 FIG S2 Transmission electron microscopy analysis of cell wall structure of planktonically grown and biofilm-associated cells of C. auris and C. albicans. Low-magnification (A to C) and high-magnification (D to F) images of planktonic cells indicating observable differences in the outer fibrillar layer with that of 0382-HBF appearing longer compared to the shorter but more dense layers seen with 0387-LBF and C. albicans. Low-magnification (G to I) and high-magnification (J to L) images of biofilm-associated cells indicated similar differences in outer fibrillar layer structure between the C. auris isolates. C. albicans biofilms consist primarily of hyphae. Bars,  500 nm (A and G) and 100 nm (B to F and H to L). Download FIG S2, TIF file, 2.6 MB. Copyright © 2020 Vila et al. 2020 Vila et al. This content is distributed under the terms of the Creative Commons Attribution 4.0 International license.

### Significantly higher C. auris recovery from infected catheters compared to C. albicans.

**FIG 3:**
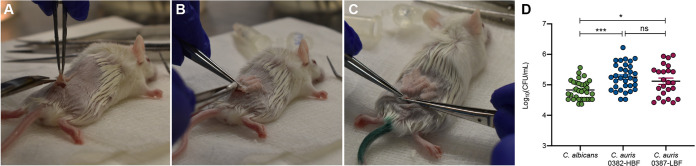
Infection and biofilm formation in catheters implanted in mice. (A) A small incision is made in a shaved area in the dorsum of anesthesized mice. (B) Up to five catheter fragments (1 cm) are inserted within a formed subcutaneous tunnel. (C) Incision is sealed with vet glue. (D) Microbial recovery from explanted catheters with 72-h biofilm formed *in vivo* demonstrating significantly higher C. auris recovery compared to C. albicans with 0382-HBF recovery being the most significant. There were no significant differences in CFU values between the C. auris isolates. Bars represent standard errors of the means. *, *P* < 0.05; ***, *P* < 0.001; ns, not significant.

Based on assessment of microbial recovery from explanted catheters, for both C. auris isolates, CFU values were significantly higher than those for C. albicans (*P* = 0.0003 for isolate 0382-HBF; *P* < 0.05 for isolate 0387-LBF) (Fig. 3D). However, although slightly higher for isolate 0382-HBF, the difference between the C. auris isolates was not significant (median values of 2 × 10^5^ and 1 × 10^5^ CFU/ml, respectively).

### Comparable levels of biofilm formation for C. auris and C. albicans within catheters by scanning electron microscopy (SEM) analysis.

**FIG 4:**
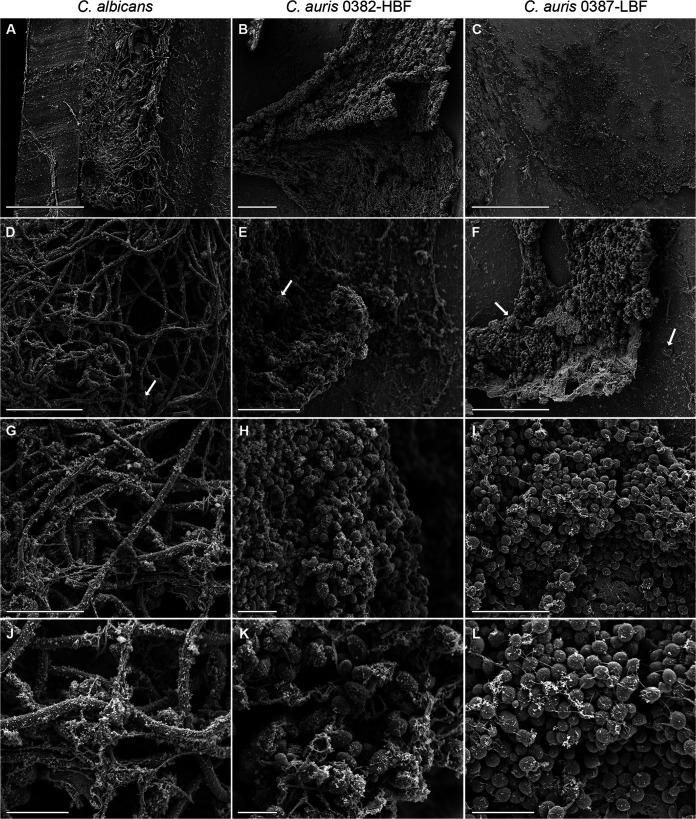
Representative low- and high-magnification scanning electron microscopy images of biofilms formed within catheters implanted in mice. Images of 72-h biofilms formed in the lumens of infected catheters harvested from mice demonstrating the formation of sheets of thick and mature biofilms for all isolates. C. albicans biofilm consisted of a matrix of hyphae and extracellular polysaccharides, and C. auris isolates formed robust multilayer biofilms consisting of yeast cells and extracellular polysaccharide matrix. SN influx of immune cells can be seen in all samples (white arrows). Bars, 200 μm (A and C), 50 μm (B, D, and F), 40 μm (E), 20 μm (G and I), 10 μm (H, J, and L), and 5 μm (K).

Representative images of catheter lumens revealed a robust and comparable ability for both C. auris isolates and C. albicans to form biofilms with extracellular matrix consisting of yeast cells for C. auris and primarily hyphae for C. albicans (Fig. 4). Influx of host immune cells can be seen in all samples (Fig. 4).

### C. auris isolates do not colonize oral tissue *in vivo*.

**FIG 5:**
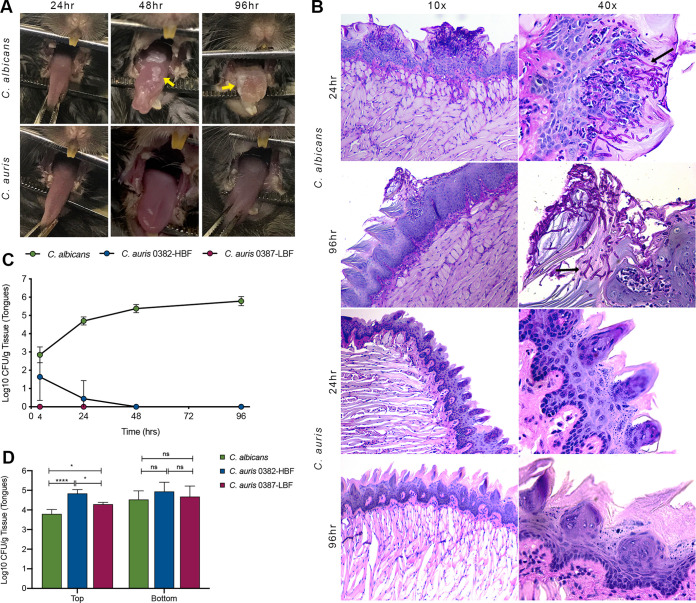
Time course evaluation of oral cavity colonization and infection in a mouse model of oral candidiasis. (A) Clinical evaluation of infected tongues. Forty-eight hours postinfection with C. albicans, white lesions indicative of advanced oral candidiasis can be seen on the dorsum of all tongues (yellow arrows). In contrast, no signs of infection or tissue inflammation were seen in any of the mice infected with C. auris (images shown for isolate 0382-HBF are representative of both isolates). (B) Histopathology analysis of infected tongue tissue. Representative low- and high-magnification images of PAS-stained tissue sections of tongues demonstrating extensive hyphal invasion of tongue epithelial tissue of C. albicans-infected animals (black arrows) with marked presence of host inflammatory cells. In contrast, tongues infected with C. auris appeared histologically healthy with no fungal presence (images shown for isolate 0382-HBF are representative of both isolates). (C) Microbial recovery from *in vivo*-infected tongues over time. Based on CFU/gram tissue weight, and consistent with clinical pictures, C. albicans recovery increased over time. In contrast, no C. auris was recovered as early as 4 h postinfection except on one occasion when isolate 0382-HBF was recovered at the 4-h time point. (D) Microbial recovery from excised tongues infected *in vitro*. In contrast to *in vivo* infection, an *ex vivo* infection model demonstrated significantly higher C. auris recovery compared to C. albicans from all tongues inoculated on the dorsum (top spiny layer) with the highest recovery for isolate 0382-HBF. However, recovery was comparable for all isolates when tongues were inoculated sublingually (bottom mucosal surface). Error bars represent standard errors of the means. *, *P* < 0.05; ****, *P* < 0.0001.

**FIG 6:**
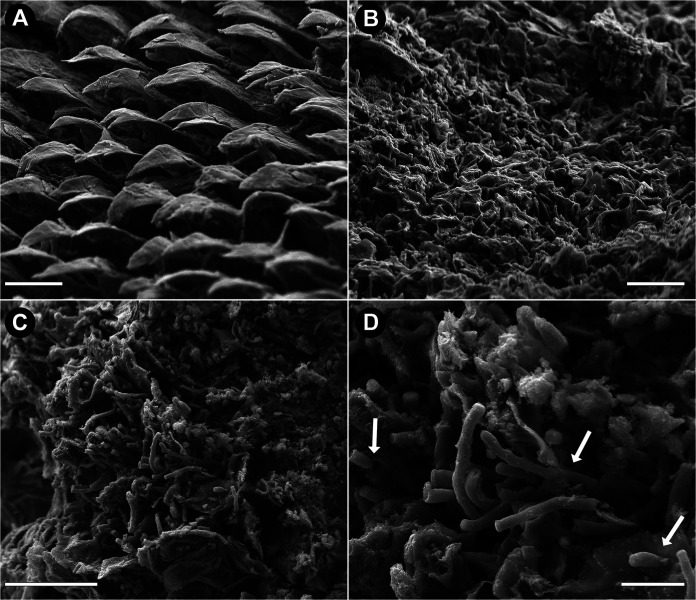
Low- and high-magnification scanning electron micrographs of the dorsum (surface) of tongues from infected mice. (A) Representative image of surface of tongue of C. auris-infected animals. No visible colonization of C. auris of either isolate was seen on the surface of any of the tongues examined. (The image shown for isolate 0382-HBF is representative of both isolates.) (B) In contrast, a thick biofilm is seen covering the surfaces of tongues from C. albicans-infected animals with clinical candidiasis. (C and D) Upon higher magnification, matrix consisting of C. albicans hyphae could be seen with the hyphae (white arrows) penetrating the epithelial tissue, causing extensive tissue damage.

CFU recovery from tongues explanted over time demonstrated increasing levels of C. albicans recovery, whereas no C. auris was recovered at any of the time points sampled (Fig. 5C). Histopathology evaluation of tongue tissue revealed extensive C. albicans hyphal penetration of the epithelium with massive influx of inflammatory cells concomitant with the presence of lesions consistent with clinical candidiasis (Fig. 5B). In contrast, for both C. auris isolates, tongues appeared healthy with no fungal presence (Fig. 5A and B). Similarly, SEM revealed a thick C. albicans matrix covering the dorsum of tongues (Fig. 6B) with hyphae penetrating epithelial layers causing extensive tissue damage (Fig. 6C). In contrast, no C. auris was seen in any sample (Fig. 6A). To demonstrate that lack of C. auris recovery from the oral infection model was not due to experimental variations in the inoculation method, *in vitro* experiments were performed to demonstrate comparable levels of uptake and release of cells from the calcium alginate swabs used for inoculating animals (Fig. S3).

10.1128/mSphere.00760-20.3 FIG S3 Control experiments for oral inoculation. To demonstrate that lack of C. auris recovery from the oral infection model was not due to experimental variations in the inoculation method, *in vitro* experiments were performed to comparatively evaluate level of uptake and release of cells from the calcium alginate swabs used for inoculating animals. As performed in infecting animals, swabs were incubated in cell suspensions for 10 min at room temperature and then either (i) placed in 100 μl PBS for 45 min (passive diffusion - representing placement of swabs in mice while under anesthesia), removed, and sample from PBS was plated or (ii) placed in 100 μl PBS for 45 min and then sonicated for 20 min and sampled from PBS plates (uptake - evaluation of inoculum in saturated swabs). Based on CFU counts, comparable level of recovery was seen for all three isolates under both experimental conditions, indicating that the inability of C. auris to colonize the oral cavity compared to C. albicans was not due to differences in inoculum delivery. A total of 12 swabs were evaluated per group on two separate occasions. Download FIG S3, TIF file, 0.1 MB. Copyright © 2020 Vila et al. 2020 Vila et al. This content is distributed under the terms of the Creative Commons Attribution 4.0 International license.

### C. auris isolates avidly adhere to tongue tissue *ex vivo*.

In stark contrast to the *in vivo* model, microbial recovery from *in vitro*-infected excised tongues demonstrated avid C. auris adherence to tissue comparable to that of C. albicans (Fig. 5D). Interestingly, compared to C. albicans, C. auris recovery was higher (*P* < 0.05) when tongues were inoculated on the dorsum spiny surface, particularly for isolate 0382-HBF (<0.0001) (Fig. 5D, left), whereas recovery was comparable for all three when tongues were inoculated sublingually.

### C. auris isolates are more adept at persisting and disseminating in an intraperitoneal infection model.

**FIG 7:**
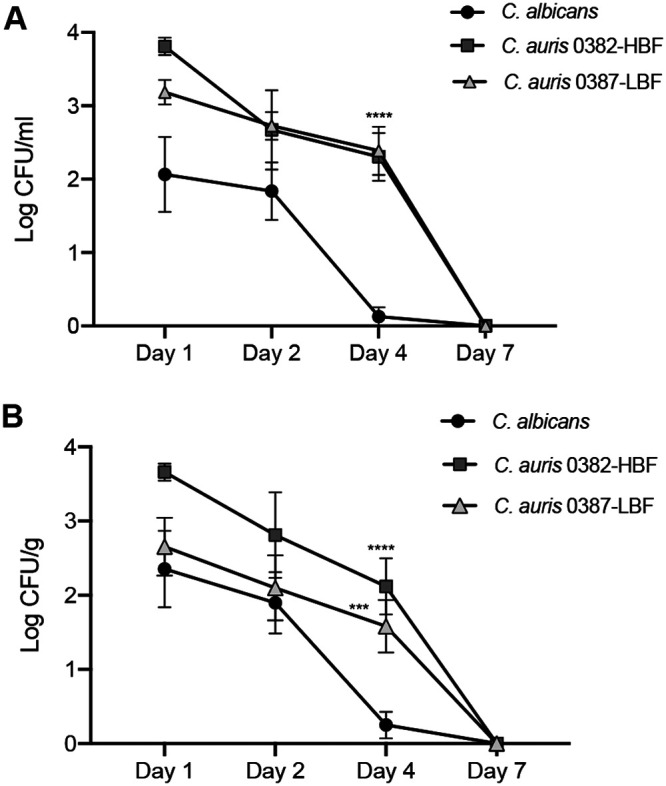
Evaluation of persistence and dissemination in a mouse model of intraperitoneal infection. (A) Microbial recovery from intraperitoneally infected mice sampled over 7 days demonstrated significantly higher and more persistent recovery for C. auris from intraperitoneal lavage fluid compared to C. albicans which was cleared from the cavity by day 4. (B) Comparable to recovery from lavage, C. auris was recovered from kidneys up to day 7, whereas C. albicans was effectively cleared by day 4. CFU for C. auris 0382-HBL were consistently higher at all time points sampled. Error bars represent standard errors of the means. ***, *P* < 0.001; ****, *P* < 0.0001.

Based on CFU/milliliter peritoneal lavage, compared to C. albicans, C. auris recovery was higher and more consistent. Time course experiments indicated C. albicans clearance from the intraperitoneal cavity 2 to 4 days postinoculation (Fig. 7A); in contrast, C. auris persisted for up to 7 days postinoculation. Similarly, C. auris was recovered from kidneys after 4 days, whereas no C. albicans was recovered after 4 days (Fig. 7B, *P* > 0.05 for day 4). Interestingly, C. auris 0382-HBF had the highest burden and dissemination potential in the first 4 days of infection (Fig. 7B).

## DISCUSSION

The multidrug-resistant species C. auris has been associated with an exponential emergence of outbreaks with life-threatening invasive disease (28, 29). In fact, C. auris is the first fungal pathogen categorized as a public health threat (30) due to its capacity to readily colonize skin and persist in abiotic surfaces of the health care environment allowing for high level of transmissibility between patients (31–34). Although relatively little is known about how C. auris colonizes and causes disease, it is quite evident that this novel pathogen is strikingly distinct among yeasts (35, 36). One unique growth feature in some isolates is cell aggregation (17) which was associated *in vitro* with differences in drug susceptibility, biofilm formation, and adhesin expression compared to nonaggregative isolates (1, 11, 14, 17, 32). Since previous studies have reported variable results for the biofilm-forming abilities of these isolates (14, 17, 18), we comparatively evaluated the 10 C. auris isolates in the CDC panel and selected the highest (0382-HBF) and lowest (0387-LBF) biofilm-forming isolates as phenotypic representatives for subsequent studies. Specifically, fluorescent confocal microscopy demonstrated a dense multilayer and homogeneous biofilm for isolate 0382-HBF, as was described for the “aggregative” phenotype, whereas isolate 0387-LBF formed a sparse biofilm with sporadic clumps of cells reported for the “nonaggregative” phenotype (18). A common observation, however, was the pronounced presence of secreted extracellular polysaccharides, which was not surprising, as large amounts of extracellular matrix were reported for C. auris biofilms *in vitro* and *in vivo* (27). These spatial-structural differences may have clinical and therapeutic implications for biofilm-associated infections, cells may easily be released from less-compact biofilms leading to disseminated disease, whereas a dense biofilm is associated with drug resistance as was described for C. albicans (37, 38).

To investigate the virulence of C. auris, several studies used the invertebrate model Galleria mellonella, and similar to the *in vitro* studies, findings have been inconsistent. In comparing C. auris to C. albicans, one study reported similar levels of virulence, while another study found the nonaggregating phenotype to be significantly more pathogenic than C. albicans (14). In another study, C. auris aggregative isolates were found to be less pathogenic than the nonaggregative isolates (17). So far, a limited number of animal studies have been performed to evaluate C. auris, with the majority using the intravenous systemic model (39–41), the most recent of which found C. auris to be less virulent than C. albicans (39). Therefore, our study was designed to comparatively evaluate the C. auris phenotypes and C. albicans using three other clinically relevant murine models of infection.

To investigate biofilm-associated infections, we used the mouse subcutaneous catheter model, a feasible and practical model to study catheter and indwelling device infections. A previous *in vitro* study evaluating C. auris adherence and biofilm formation on catheters found C. auris to adhere less to silicon elastomer catheters than C. albicans (13, 42); however, based on microbial recovery from explanted catheters, we found recovery of both C. auris isolates to be significantly higher than that of C. albicans when biofilms were formed *in vivo*. Although surprising, the seemingly superior adherence of C. auris to catheter material may explicate the emergence of C. auris as a nosocomial pathogen, given that catheter infections are considered a predictor for invasive C. auris infection in hospitalized patients (10, 29). The ability of C. auris to adhere to catheters and form biofilm *in vivo* was also demonstrated by SEM analysis revealing a massive amount of extracellular biofilm matrix, consistent with what was observed in a rat model of central-venous-catheter infection (27).

C. albicans is a common colonizer of the oral mucosal surfaces and the causative agent of oral candidiasis (43, 44); however, the ability of C. auris to adhere and colonize oral mucosal tissue has not been investigated. Using a modification of our established mouse model (45), we performed comparative time course studies to monitor colonization and disease development over time. Our findings demonstrated that both C. auris strains were cleared from the oral cavity within 4 h of inoculation, whereas C. albicans persisted, causing advanced clinical candidiasis. As hyphae are responsible for tissue penetration and mucosal infection, C. auris was not expected to cause clinical disease; however, the complete inability to colonize oral tissue was somewhat surprising. To explore whether this was due to deficiency in tissue adherence, we performed *ex vivo* experiments on excised mouse tongues. Surprisingly, in contrast to the *in vivo* findings, both C. auris isolates were recovered in higher numbers than C. albicans. C. albicans adherence to surfaces is mediated by glycosylphosphatidylinositol (GPI)-linked cell wall adhesins, most notably the ALS (agglutinin-like sequence) family of glycoproteins, with Als3 being the key member for tissue adherence (46). Interestingly, in a C. auris transcriptome analysis of *in vitro*-grown biofilms, Kean et al. (47) found only *ALS1* and *ALS5*. However, using bioinformatic and structural homology modeling, Singh et al. (48) found protein homologs of Ca-Als3p for C. auris and demonstrated *in vivo* humoral and cell-mediated protection upon vaccination with anti-Als3p antibodies. Nevertheless, the lack of key adhesins may explain the inability of C. auris to colonize the oral cavity. Alternatively, although speculative at this juncture, the discrepancies in C. auris tissue adherence under *ex vivo* and *in vivo* conditions suggest a key role for the host in controlling C. auris colonization. The contribution of host immune factors cannot be disregarded, as *in vitro* studies have indicated significant differences in host response to C. auris isolates. Using the zebrafish model, Johnson et al. (49) demonstrated that C. auris cells are able to avoid neutrophil recognition and survive in neutrophils upon phagocytosis. Interestingly, Pathirana et al. (18) found that strain 0382-HBF is able to survive inside neutrophils significantly better than C. albicans, whereas strain 0387-LBF was susceptible to neutrophil killing. Additionally, unique fungal recognition and/or host profiles to different C. auris phenotypes were also reported using an *in vitro* skin wound model (1).

Using the mouse model of intraperitoneal infection, previous studies showed C. albicans is effectively cleared from the peritoneal cavity of immunocompetent mice (50). Similarly, in our hands, C. albicans was cleared 2 to 4 days postinfection. In contrast, C. auris isolates persisted and were recovered from kidneys for up to 7 days postinfection. Although warranting further investigations, these preliminary findings are in line with *in vitro* findings demonstrating that human neutrophils preferentially engage and kill C. albicans over C. auris (49) and that innate immune recognition of C. auris and phagocytosis are different from those for C. albicans (51). Interestingly, Torres et al. (41) demonstrated that neutrophil-depleted mice succumb to systemic C. auris infection, while very small amounts of C. auris persisted in the immunocompetent infected control without causing disease, suggesting a potential opportunistic resurgence upon a change in immune status (41). Combined with our studies, these studies strongly suggest that C. auris employs unique strategies to interact with and infect the host in various niches.

In summary, in this study, we used three different animal models to comparatively evaluate the host site-specific pathogenic potential of C. auris. The findings from the catheter infection model demonstrated the avidity of C. auris to adhere and form robust biofilms on abiotic surfaces supporting the strong association of C. auris systemic diseases with the presence of indwelling catheters. Importantly, we demonstrate for the first time the inability of C. auris to colonize the oral cavity in a host, attributing a potential role for efficient host immune clearance on mucosal surfaces. In contrast, in the intraperitoneal infection model, C. auris persisted longer in the peritoneal cavity and kidneys. Interestingly however, although there were clear niche-specific differences in pathogenic features between C. auris and C. albicans, overall we did not observe significant differences between the C. auris phenotypes in the animal models. One limitation of the study is that only the C. albicans standard strain SC5314, which displays high virulence and biofilm formation, was used for comparison. Nevertheless, the combined findings warrant further in-depth analysis into the unique virulence traits of C. auris and the niche-specific host-pathogen interactions. A clear understanding of the various host and pathogen factors that have contributed to the rise of C. auris as a nosocomial pathogen is critical for developing new strategies to prevent and control the spread of this multidrug-resistant pathogen.

## MATERIALS AND METHODS

### *In vitro* biofilm formation.

The reference strain C. albicans SC5314 (52) and the C. auris CDC panel containing 10 C. auris isolates (Antibiotic Resistance [AR] Isolate Bank number 0381-0390) were used in this study. Isolates were grown overnight in yeast peptone dextrose broth (YPD) (Difco Laboratories) at 30°C, washed in phosphate-buffered saline (PBS), and resuspended in RPMI 1640-HEPES (Invitrogen) medium (1 × 10^6^ cells/ml). For comparative evaluation of biofilm formation, C. auris isolates and C. albicans biofilms were grown by seeding 100 μl of cell suspensions in flat-bottom 96-well polystyrene microtiter plates. Following incubation at 37°C for 24 h, the wells were washed with PBS and biofilms were evaluated using the 3-(4,5-dimethylthiazol-2-yl)-5-(3-carboxymethoxyphenyl)-2-(4-sulfophenyl)-2H-tetrazolium (MTS) metabolic assay (Promega) per the manufacturer’s recommendation. Color intensity was measured at 490 nm using a cell imaging multimode reader (Cytation 5; Biotek). Biofilms were also simultaneously evaluated by phase-contrast imaging. Based on initial evaluations, two C. auris strains representing “high biofilm former” (0382-HBF) and “low biofilm former” (0387-LBF) phenotypes were selected for subsequent experiments. Both isolates belong to clade II (East Asian) and were isolated in Pakistan; isolate 0382 was recovered from a burn wound, and isolate 0387 was recovered from blood.

### Confocal laser scanning microscopy (CLSM) evaluation of *in vitro*-grown biofilms.

C. auris 0382-HBF and 0387-LBF and C. albicans biofilms were grown in 24-well glass-bottom microtiter plates for 24 h as described above; biofilms were stained with a concanavalin A-conjugated to Alexa Fluor 647 (Invitrogen) (50 μg/ml) for 45 min at 37°C and 0.1% calcofluor stain (Sigma-Aldrich) for 10 min at room temperature. Biofilms were visualized using an inverted confocal laser scanning microscope (T2i; Nikon), and images were analyzed using Imaris 9.3 Arena software and ImageJ.

### Transmission electron microscopy evaluation of cell ultrastructure.

Cells harvested from biofilms and planktonic cultures were fixed and embedded in agarose, and blocks were postfixed with 1% osmium tetroxide−1.5% potassium ferrocyanide and then stained with uranyl acetate. Specimens were serially dehydrated in ethanol and embedded in Spurr resin. Ultrathin sections (∼70 nm) were examined with a Tecnai T12 transmission electron microscope (TEM) (Thermo Fisher Scientific), and images were processed using Adobe Photoshop software.

### Animal studies.

All animal experiments were conducted at the AAALAAC-accredited Animal Facility of the University of Maryland, Baltimore, and were approved by Animal Care and Use Committee. Three-month-old female C57BL/6 mice (oral model) and BALB/c mice (catheter and intraperitoneal models) were purchased from Envigo. Mice were housed at a maximum of five per cage, weighed, and closely monitored for any signs of distress throughout experimental periods.

### Mouse subcutaneous catheter infection model.

The model previously described by Kucharíková et al. (53) was used with modifications. Fragments (0.5 cm) of polyurethane triple-lumen central venous catheters (Jorgensen Laboratories) precoated overnight with fetal bovine serum (Gibco) were incubated with 1 × 10^8^ cells/ml cell suspensions in RPMI for 1.5 h at 37°C, rinsed, and kept on ice until implanted. BALB/c mice were anesthesized with 0.5 ml intraperitoneal injections of tribromoethanol (TBE) solution (250 mg/kg of body weight; Sigma-Aldrich); the dorsum was shaved, a small incision was made aseptically, and a subcutaneous tunnel was created allowing for insertion of five to eight pieces of preinoculated catheters (Fig. 3A to C). Incisions were sealed using 3M Vetbond tissue glue, and lidocaine analgesic gel was applied. Biofilms were allowed to form within catheters for 72 h, and then animals were euthanized by CO_2_ inhalation followed by cervical dislocation. Catheters were harvested individually, aseptically fragmented, and sonicated in sterile PBS to detach biofilms. Cell suspensions were diluted and plated in triplicate on chromogenic medium CHROMagar (DRG International) for CFU enumeration. A total of seven mice were included for each group, and a total of 25 to 35 catheters were analyzed individually. Experiments were performed on four separate occasions, and the data were combined.

### Scanning electron microscopy of explanted catheters.

From each group, representative catheters were processed for scanning electron microscopy (SEM) to visualize *in vivo*-grown biofilms. Catheters were cut longitudinally to expose the lumen, fixed in 2% paraformaldehyde−2.5% glutaraldehyde, postfixed with 1% osmium tetroxide, serially dehydrated in ethyl alcohol (30 to 100%), and critical point dried. Samples were coated with carbon and observed with Quanta 200 SEM (FEI Co.), and images were processed using Adobe Photoshop software.

### Mouse model of oropharyngeal candidiasis.

The established mouse model of oropharyngeal candidiasis (54) was used as we previously performed (45). C57BL/6 mice were immunocompromised by subcutaneous administration (0.2 ml; 250 mg/kg) of cortisone-acetate (Sigma-Aldrich) in the neck dorsum every other day starting 1 day preinfection. Animals were divided into three groups for infection with (i) C. albicans, (ii) C. auris 0382-HBF, and (iii) C. auris 0387-LBF. On the day of infection, mice were anesthetized with TBE and then orally infected by placing calcium alginate swabs (Fisher Scientific, Waltham, MA) saturated (10 min, room temperature [RT]) with cell suspension (2 × 10^7^ cells/ml). Swabs were kept sublingually for 45 min, and the animal’s body temperature was maintained at 37°C using a heat lamp. Animals were monitored until they recovered from anesthesia and daily for any clinical signs of distress Animals were euthanized at different time points (4, 24, 48, and 96 h postinfection), and their tongues were harvested, weighed, homogenized, and cultured in triplicate on CHROMagar for CFU enumeration (CFU/gram tissue weight). A total of three or four mice were included in each group per time point; experiments were performed on two separate occasions, and results were combined.

### Histopathology and SEM of infected tongues.

Representative tongues from each group were fixed in 10% formalin and embedded in paraffin, and xylene-deparaffinized sections were stained with periodic acid-Schiff (PAS); slides were examined by light microscopy. Representative tongues were also processed for SEM as described above.

### Tongue *ex vivo* model of infection.

An *ex vivo* infection model was used as we previously described (55) to specifically evaluate tissue adherence abilities in the absence of host immune factors. Tongues excised from healthy euthanized mice were weighed and infected *in vitro* mimicking *in vivo* procedure; top or bottom surfaces of tongues were streaked with saturated swabs which were then left under the tongues for 45 min at 37°C. Tongues were rinsed, homogenized in PBS, and plated for CFU enumeration.

### Mouse model of intraperitoneal infection.

BALB/c mice were injected intraperitoneally with 0.2 ml of cell suspensions (3.5 × 10^7^ cells/ml), and groups of animals were euthanized at different time points: 1, 2, 4, and 7 days postinfection. Peritoneal cavities were lavaged by injection of 3 ml sterile PBS followed by gentle massaging; fluid was carefully recovered with a syringe. Kidneys were harvested, weighed, and homogenized for evaluation of disseminated disease. Lavage fluid and kidney homogenates were diluted and plated for CFU enumeration. A total of 29 mice were included in each group. The experiments were performed on three separate occasions, and the results were combined.

### Data analysis.

Statistical analysis was performed using GraphPad Prism 8.0 software. The standard error of the mean was used in all graphs; one-way analysis of variance (one-way ANOVA) was used with Tukey’s *posthoc* test. *P* values of <0.05 were considered significant.
