# Supplementary figures and images for: Comparative Evaluations of the Pathogenesis of Candida auris Phenotypes and Candida albicans Using Clinically Relevant Murine Models of Infections
Source: mSphere. 2020 Aug 5;5(4):e00760-20. doi: 10.1128/mSphere.00760-20 (PMC7407074; doi:10.1128/mSphere.00760-20)

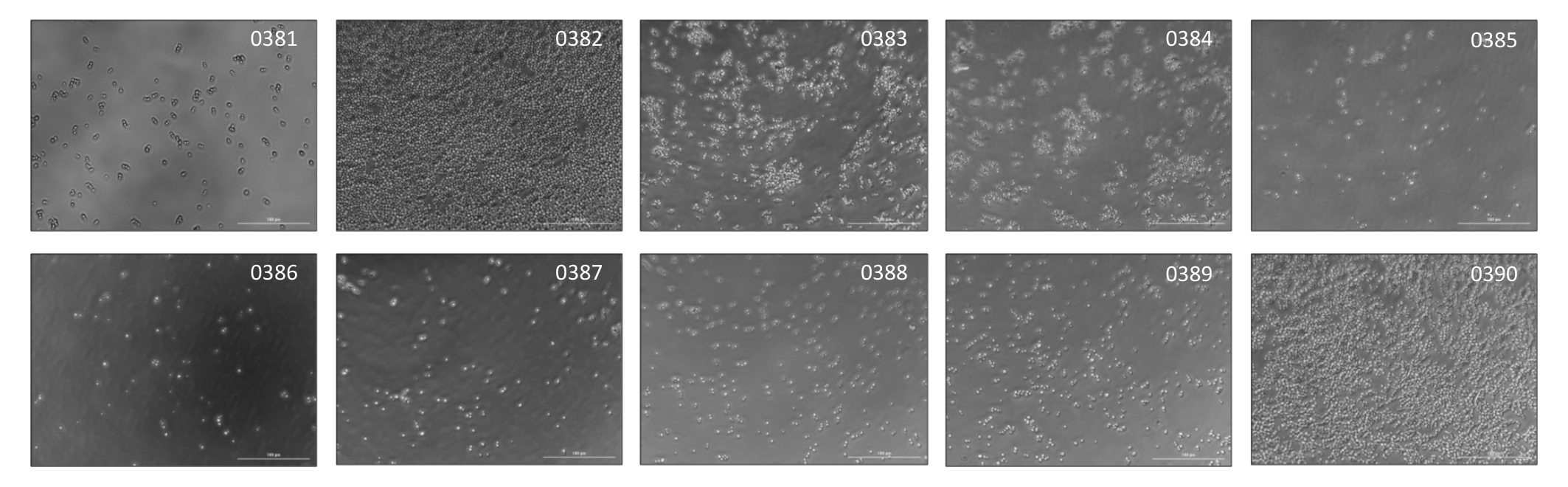

Supplement: FIG S1 [file mSphere.00760-20-sf001.tif]

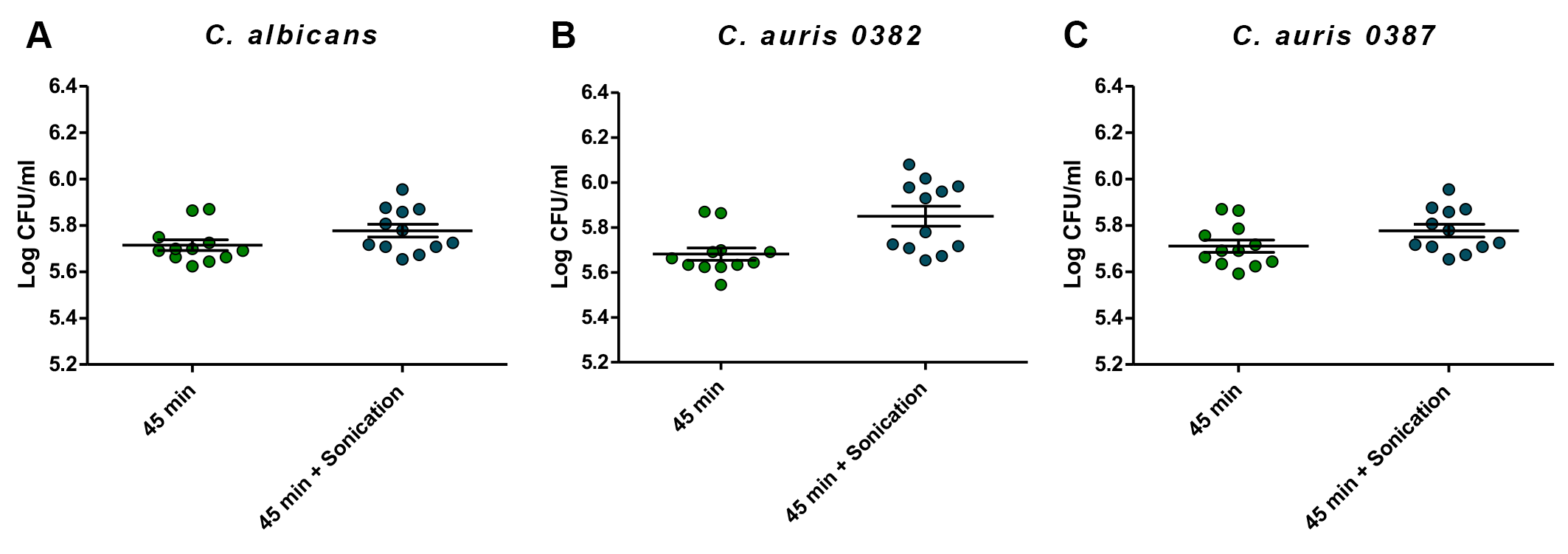

Supplement: FIG S3 [file mSphere.00760-20-sf003.tif]
